# An in vitro study on sonodynamic treatment of human colon cancer cells using sinoporphyrin sodium as sonosensitizer

**DOI:** 10.1186/s12938-020-00797-w

**Published:** 2020-06-17

**Authors:** Yuanyuan Shen, Jianquan Ou, Xin Chen, Xiaojun Zeng, Lanhui Huang, Zhaoke Pi, Yaxin Hu, Siping Chen, Tie Chen

**Affiliations:** 1 National-Regional Key Technology Engineering Laboratory for Medical Ultrasound, School of Biomedical Engineering, Health Science Center, Shenzhen University, Shenzhen, Guangdong People’s Republic of China; 2 Department of Pharmacy, Health Science Center, Shenzhen University, Shenzhen, Guangdong People’s Republic of China; 3 Shenzhen Second People’s Hospital, Shenzhen, People’s Republic of China

**Keywords:** Sonodynamic therapy, Sinoporphyrin sodium, Colorectal cancer

## Abstract

**Background:**

Colorectal cancer is the third leading cause of cancer-related deaths worldwide. Sonodynamic therapy (SDT) is an emerging cancer therapy, and in contrast to photodynamic therapy, could non-invasively reach deep-seated tissues and locally activates a sonosensitizer preferentially accumulated in the tumor area to produce cytotoxicity effects. In comparison with traditional treatments, SDT may serve as an alternative strategy for human colon cancer treatment. Here, we investigated the sonodynamic effect using sinoporphyrin sodium (DVDMS) as a novel sonosensitizer on human colon cancer cells in vitro.

**Results:**

The absorption spectra of DVDMS revealed maximum absorption at 363 nm wavelength and emission peak at 635 nm. Confocal microscopy images revealed the DVDMS was primarily localized in the cytoplasm, while no evident signal was detected within the nuclei. Flow cytometry analysis showed rapid intracellular uptake of DVDMS by two types of human colon cancer cells (HCT116 and RKO). Cell viability of HCT116 was tolerant with the concentration of DVDMS up to 20 µg/mL, while the case of RKO was 5 µg/mL. In comparison with the control group, the SDT-treated groups of these two types of human colon cancer cells showed significant increase in cellular apoptosis and necrosis ratio. Increased intracellular reactive oxygen species (ROS) production was detected, indicating the involvement of ROS in mediating SDT effects.

**Conclusion:**

DVDMS results an effective sonosensitizer for the ultrasound-mediated cancer cell killing, and its anticancer effect seems to rely on its ability to produce ROS under ultrasound exposure.

## Background

Colorectal cancer (CRC) is one of the most common tumors among both men and women worldwide. The incidence and mortality of CRC remain a serious problem, especially in developed countries [1]. Traditional treatment options such as surgery, radiation therapy, and chemotherapy have severe side-effects, necessitating the development of novel treatment regimens for CRC [2].

Sonodynamic therapy (SDT) has evolved as a promising therapeutic approach for cancer treatment over recent decades. This technique involves the cytotoxic effects elicited by non-toxic chemical agents preferentially retained in tumor tissue upon exposure to relatively low-intensity ultrasound. Serving as sonosensitizers, the sono-responsive chemicals combined with ultrasound offer advantages of minimizing adverse effects and maximizing on-target responses, particularly for the non-invasive treatment of less-accessible cancers [3, 4]. The ultrasound intensity used for SDT is relatively low, facilitating penetration into deeply seated tumor tissues as compared with photodynamic therapy (PDT), which has limited efficacy owing to the use of laser light [5]. During the last decade, SDT has been efficiently employed for the treatment of human colon cancer in vitro and in vivo. Studies have investigated the effectiveness of high energy shock wave (HESW) combined with 5-aminolevulinic acid (ALA) as the sonosensitizer for the treatment of HT-29 human colon adenocarcinoma cells [6] and DHD/K12/TRb (PROb) rat colon adenocarcinoma cells [7]. A remarkable improvement in cell inhibition effect was observed upon exposure to SDT. The antitumor effects of SDT with several types of sonosensitizers were also investigated in mice bearing colon 26 carcinoma [8, 9]. These studies found that the tumor sizes significantly decreased with an increase in the dosage of sensitizers at an acoustic intensity of 3 W/cm^2^. In addition, it was reported that SDT mediated by gold nanoparticles in conjugation to protoporphyrin IX (PpIX) could reduce the volume of colon carcinoma tumors and prolong the survival time of tumor-bearing mice [10]. These results imply that SDT may be potentially useful for the treatment of human colon cancer.

Sonosensitizers are vital components of SDT. The physical and chemical properties of a sonosensitizer could not only influence the therapeutic effect of SDT but also determine the safety of drug residue in the human body. Many photosensitizers such as hematoporphyrin, photofrin II, ATX-70, ZnPcS_2_P_2_, and protoporphyrin IX also act as sonosensitizers and induce strong antitumor effects in SDT [11]. Recently, a novel chemical agent sinoporphyrin sodium (DVDMS), depurated from photofrin II, has gained increasing attention [12–18]. In comparison with photofrin II, DVDMS is highly pure and water soluble with low skin phototoxicity and produces high levels of reactive oxygen species (ROS) [13, 14, 19]. It has been reported that the molar extinction coefficient of DVDMS was much higher at 405 or 630 nm, about one order of magnitude greater than that of Photofrin [20]. This extremely high extinction coefficient probably results in the high singlet oxygen generation efficiency of DVDMS. Apart from being an effective photosensitizer, DVDMS is also sono-responsive to induce cytotoxicity upon exposure to an acoustic field [21]. DVDMS was found to preferentially accumulate in sarcoma 180 solid tumors and could be locally activated by ultrasound to non-invasively elicit a strong cytotoxic effect without causing any damage to adjacent tissues [16]. In comparison with hematoporphyrin, DVDMS-mediated SDT was more cytotoxic to ECA-109 cells in vivo, eliciting severe mitochondrial damage and high ROS production [17]. In addition, our previous study investigated the anticancer effects of DVDMS-mediated SDT against human glioblastoma cancer in vitro and in vivo. Apoptosis induction and cell proliferation suppression markedly increased by SDT after the enhanced delivery of DVDMS by ultrasound and microbubbles [18]. However, to the best of our knowledge, studies describing the synergistic effects of ultrasound and DVDMS on human colon cancer in vitro or in vivo are extremely rare.

Here, we evaluated the sonodynamic effects of DVDMS as a sonosensitizer on two types of human colon cancer cells (HCT116 and RKO) in vitro. Ultraviolet spectrophotometer system and microplate reader were used to analyze spectral characteristics of DVDMS, and Cell Counting Kit 8 (CCK8) assay was adopted to investigate its cytotoxicity. The intracellular localization of DVDMS was imaged with a laser scanning confocal microscope. The time-dependent accumulation of DVDMS was observed with flow cytometry and microplate reader, while DVDMS-mediated cellular apoptosis and necrosis were analyzed with fluorescein isothiocyanate (FITC) and propidium iodide (PI) double staining. Fluorescence microscopy and flow cytometry were used to assess the generation of ROS.

## Results

### Spectral analysis of DVDMS

**Fig. 1:**
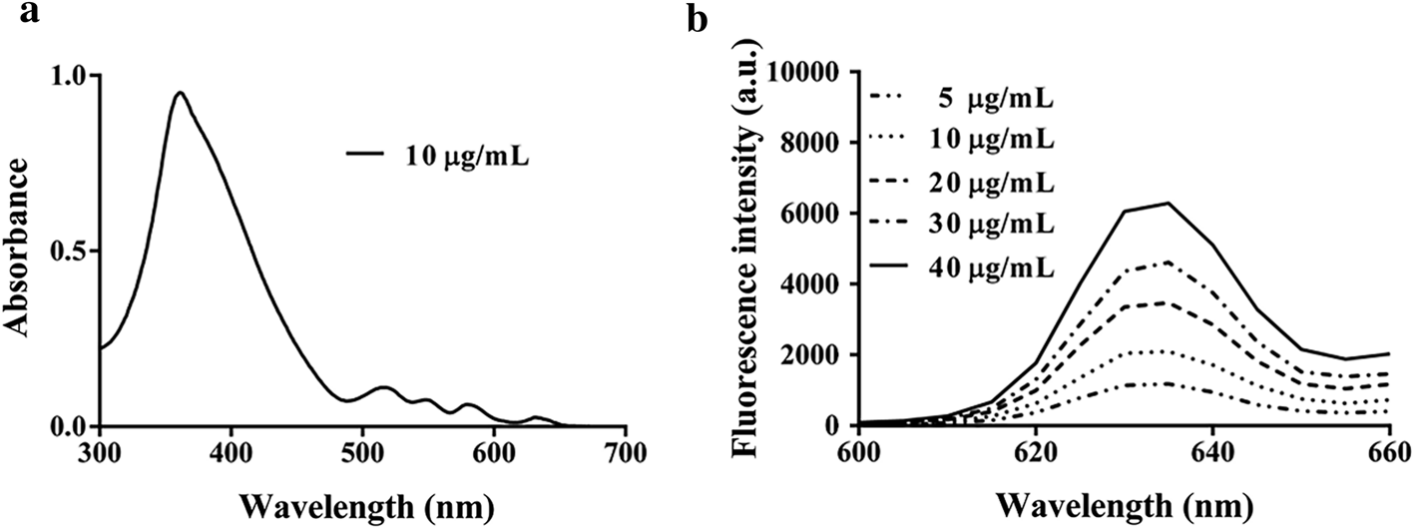
Spectral analysis of DVDMS. **a** Absorption spectra of DVDMS. **b** Emission spectra of DVDMS

The spectral characteristics of DVDMS were analyzed at different concentrations in the range of 5 to 40 µg/mL. As a result, five distinct absorption peaks were recorded at 360, 516, 548, 580, and 632 nm. The peak with maximum absorption was at 363 nm (Fig. 1a). The results of emission spectra showed that the optimum fluorescence emission peak was at 635 nm (Fig. 1b). The shape and peak wavelength of the emission spectrum were independent of concentration.

### Cytotoxicity analysis of DVDMS

**Fig. 2:**
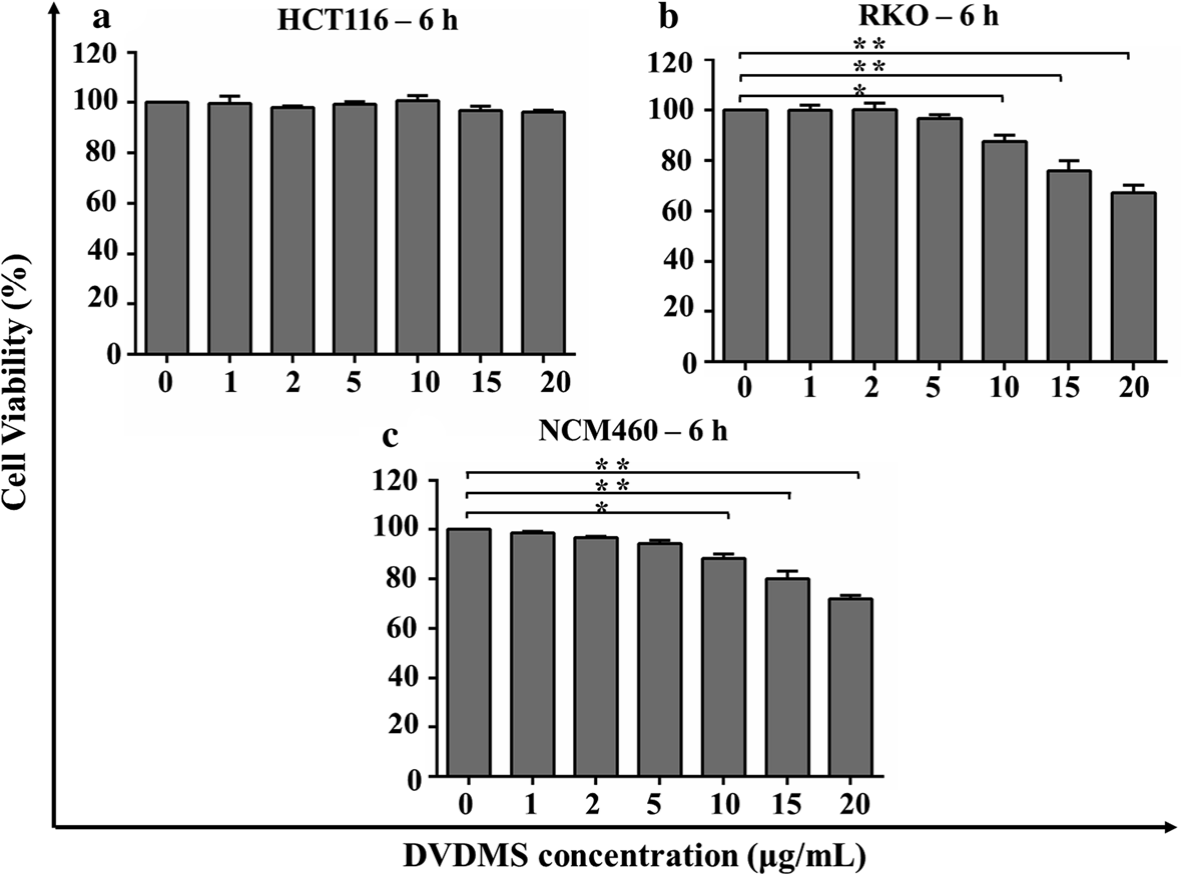
Viability of human colon cancer cells and normal colon cells after incubation with DVDMS at different concentrations for 6 h. **a** HCT116 cells, **b** RKO cells and **c** NCM460 cells. *p < 0.05 versus control. **p < 0.01 versus control

To examine the cytotoxicity of DVDMS, the viability of human colon cancer cells (HCT116, RKO) and normal colon cells (NCM460) incubated with various concentrations of DVDMS (0, 1, 2, 5, 10, 15, and 20 µg/mL) for 6 h was analyzed. As shown in Fig. 2a, more than 96.10 ± 0.76% of HCT116 cells were viable after incubation with DVDMS at concentrations up to 20 µg/mL. The viability of HCT116 cells showed no dependency on DVDMS dose. However, after treatment with DVDMS at concentrations up to 10 µg/mL, the viability of RKO (Fig. 2b) cells and NCM460 cells (Fig. 2c) began to decrease and dropped to 87.54 ± 2.50% (*p* < 0.05 versus control) and 88.23 ± 1.81% (*p* < 0.05 versus control), respectively. The cell viability at 20 µg/mL concentration was only 67.22 ± 3.02% (*p* < 0.01 versus control) and 71.92 ± 1.4% (*p* < 0.01 versus control), respectively. Based on these findings, we chose 5 µg/mL DVDMS concentration for subsequent experiments.

### Intracellular uptake of DVDMS

**Fig. 3:**
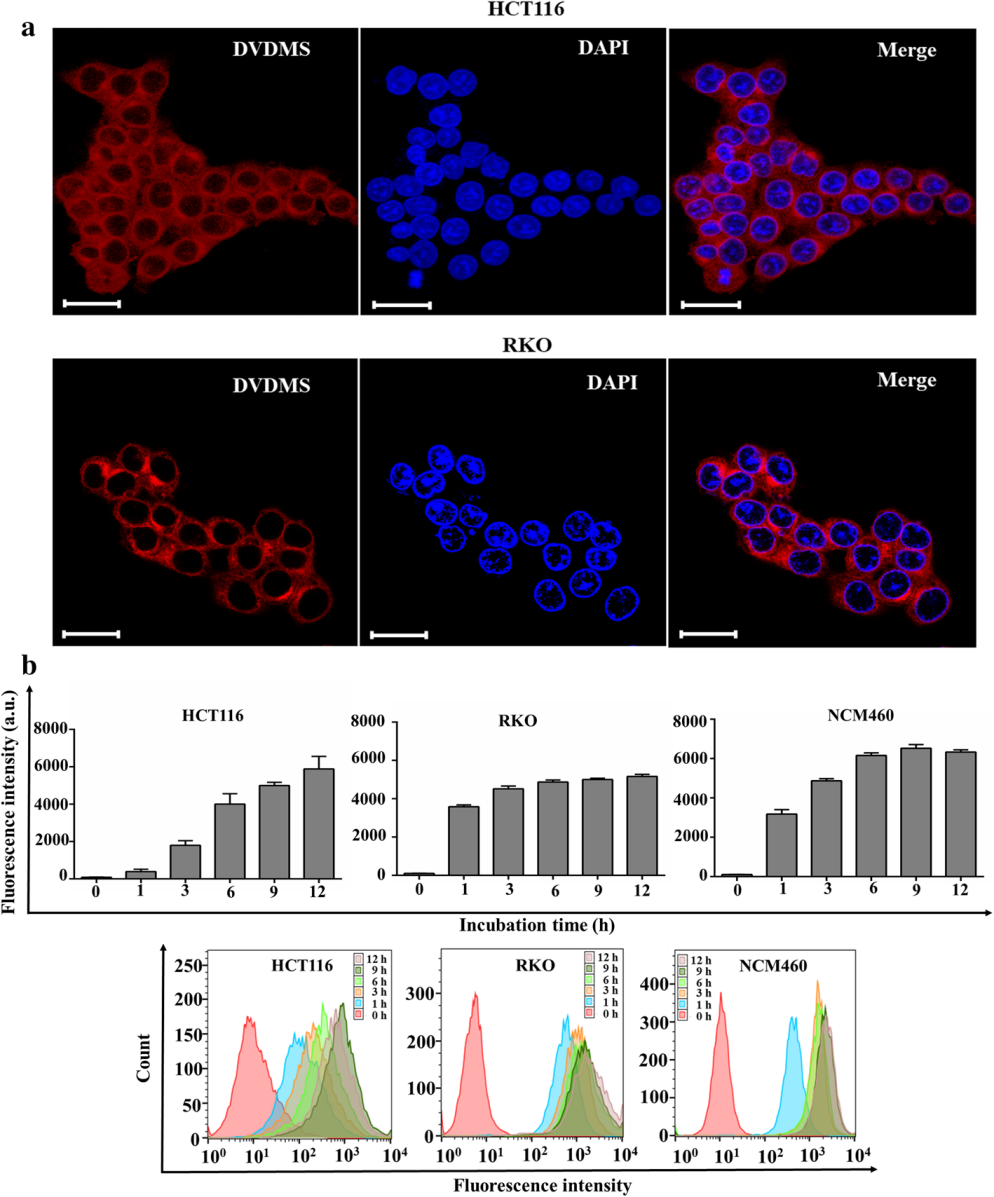
Intracellular uptake of DVDMS. **a** Intracellular localization of DVDMS (red) in HCT116 cells and RKO cells. The nuclei were stained with DAPI (blue). Scale bar 50 µm. **b** Measurement of fluorescence intensity of intracellular DVDMS in HCT116, RKO and NCM460 cells with different incubation durations by a microplate reader and flow cytometry

The intracellular uptake of DVDMS by HCT116 and RKO cells were examined by imaging the fluorescence signal of DVDMS under a confocal microscope. As shown in Fig. 3a, the internalization of DVDMS could be observed in most HCT116 and RKO cells. Besides, DVDMS was primarily localized in the cytoplasm, while no evident signal was detected within the nuclei. The fluorescence intensity of DVDMS in HCT116, NCM460, and RKO cells with different incubation times was measured with a microplate reader and flow cytometry. As shown in Fig. 3b, the fluorescence intensity of DVDMS in the two types of human colon cancer cells was much higher when the incubation lasted more than 6 h. Thus, we chose 6 h as the optimal incubation time for SDT treatment. Similar to human colon cancer cells, the internalization of DVDMS by normal colon cells NCM460 also increased significantly with the increase of incubation duration.

### SDT-mediated apoptosis and necrosis

**Fig. 4:**
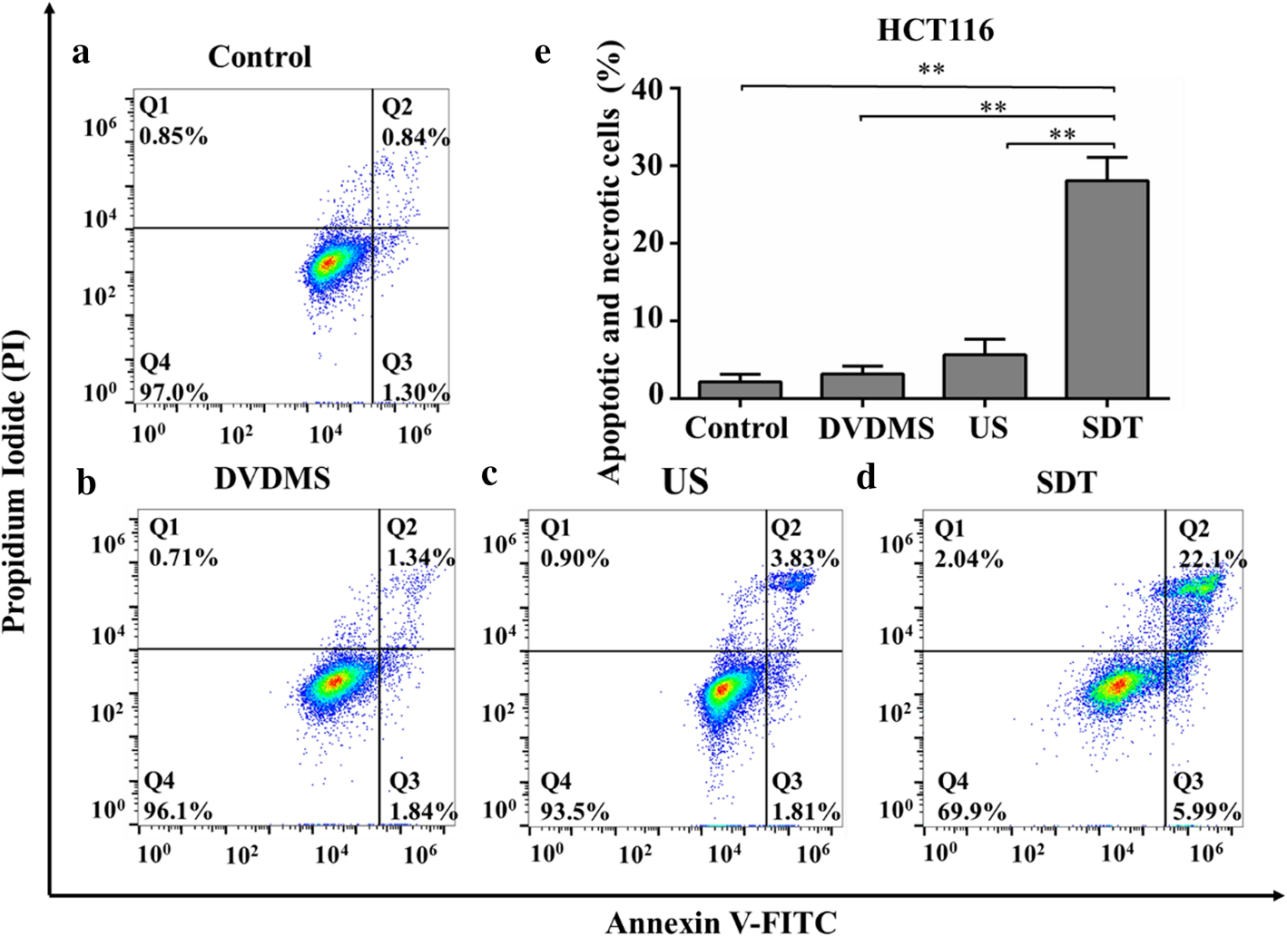
Apoptosis and necrosis analyses of HCT116 cells exposed to different treatment regimens. Representative flow cytometry results of cells at 2 h after exposing to **a** no treatment, **b** DVDMS (5 µg/mL) alone, **c** ultrasound alone, and **d** SDT using DVDMS (5 µg/mL). **e** The ratio of apoptotic and necrotic cells in four groups. **p < 0.01 versus control, DVDMS, and US groups

**Fig. 5:**
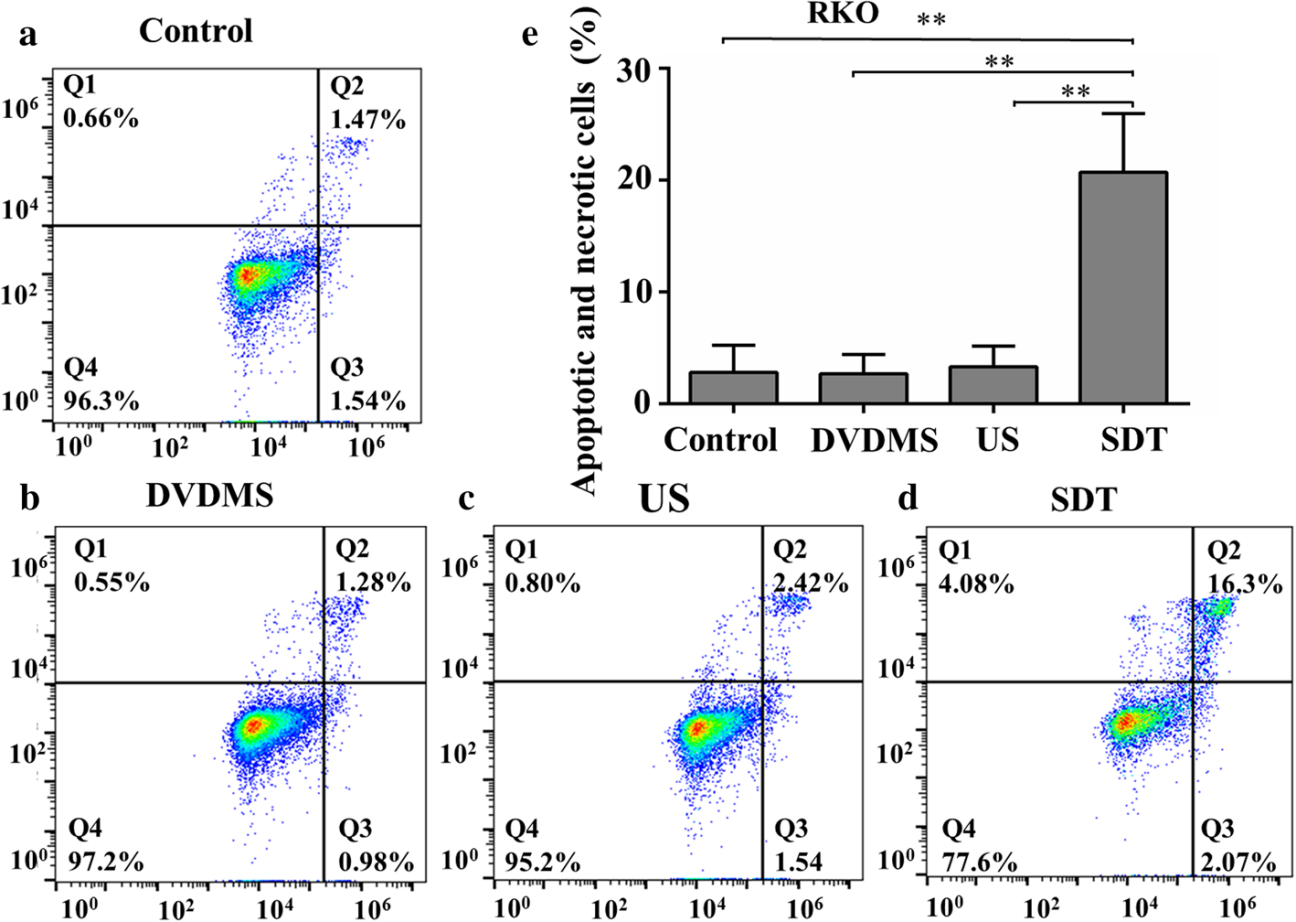
Apoptosis and necrosis analyses of RKO cells exposed to different treatment regimens. Representative flow cytometry results of cells at 2 h after exposing to **a** no treatment, **b** DVDMS (5 µg/mL) alone, **c** ultrasound alone, and **d** SDT using DVDMS (5 µg/mL). **e** The ratio of apoptotic and necrotic cells in four groups. **p < 0.01 versus control, DVDMS, and US groups

Cellular apoptosis and necrosis were evaluated with flow cytometry at 2 h after different treatments using FITC and PI staining. As shown in Figs. 4 and 5, the cells from the Q1 quadrant represent necrotic cells, while those from Q2 and Q3 quadrants indicate apoptotic cells. Viable cells appear in the Q4 quadrant. Apoptosis and necrosis in HCT116 cells after SDT treatment showed 96.32 ± 1.44%, 95.65 ± 0.79%, and 94.37 ± 1.83% viable cells in control, DVDMS, and US groups, respectively (Fig. 4). The cell apoptosis ratio for DVDMS (3.45 ± 1.27%) and the US (5.64 ± 2.31%) groups was not significantly different from that in the control group (2.24 ± 0.9%). In addition, only 0.86 ± 0.78%, 0.76 ± 0.61%, and 1.03 ± 0.33% of necrotic cells were detected in the control, DVDMS, and US group, respectively. However, in the SDT group, the percentage of apoptotic and necrotic cells significantly increased at a ratio of 29.09 ± 3.12% (*p* < 0.01 versus control) and the ratio of viable cells decreased to 68.6 ± 3.4% (Fig. 4e). Similarly, the evaluation of apoptosis and necrosis in RKO cells showed 95.90 ± 0.57%, 97.00 ± 0.29%, and 95.84 ± 0.66% viable cells in the control, DVDMS, and US groups, indicating no significant differences among them (Fig. 5). In contrast, the ratio of apoptotic and necrotic cells in the SDT group significantly increased to 20.76 ± 1.89% (*p* < 0.01 versus control). These results revealed the effectiveness of SDT using DVDMS as sonosensitizer for the treatment of human colon cancer cells in vitro.

### Intracellular ROS evaluation

**Fig. 6:**
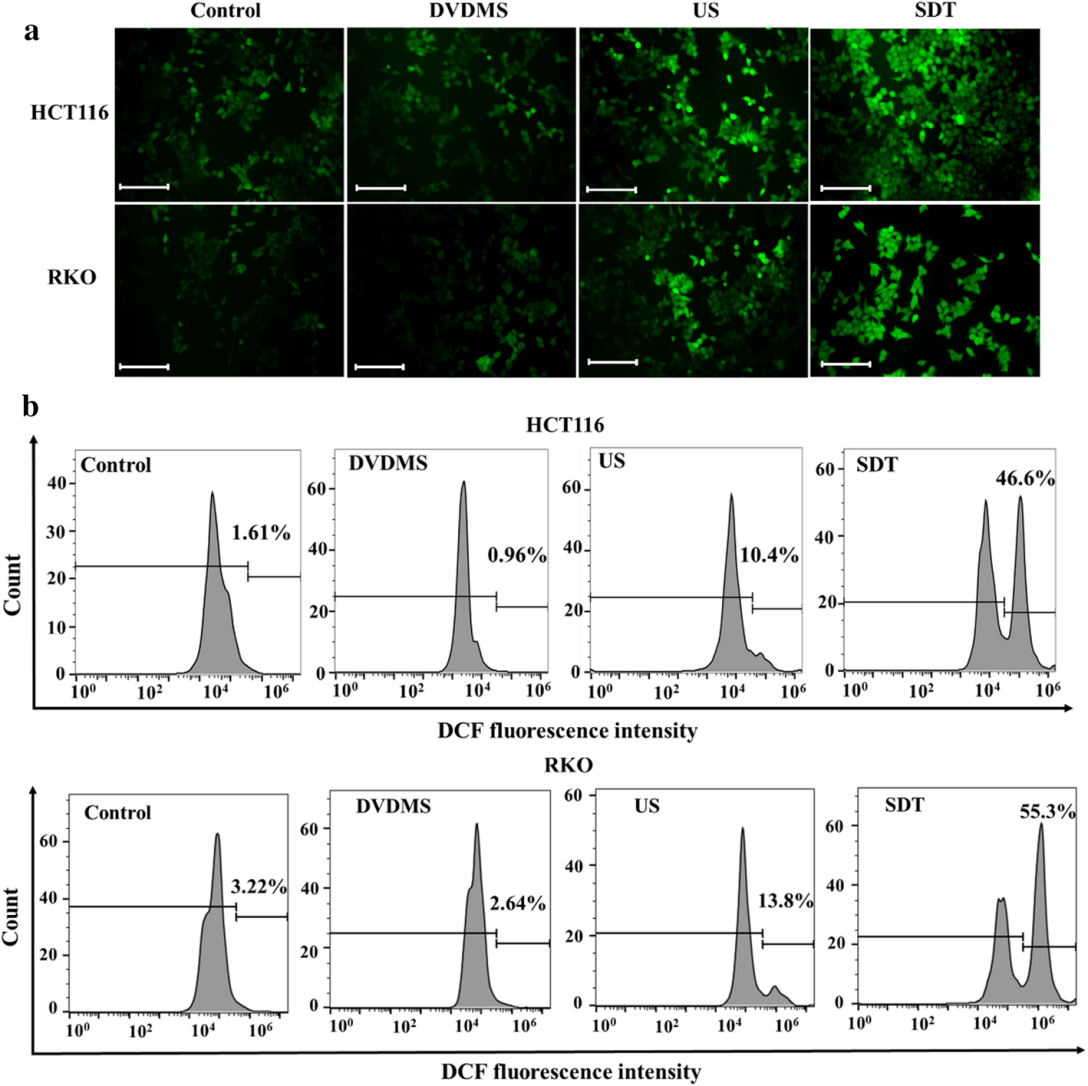
Intracellular ROS level of HCT116 and RKO cells in four groups. After SDT treatment, the cells were cultured in the dark for additional 2 h before ROS analyses. **a** Representative fluorescence images of DCF (green), probing intracellular ROS. **b** Representative histogram of fluorescence intensity of DCF in HCT116 and RKO cells detected by flow cytometry. Scale bar 100 µm

Intracellular ROS level was investigated using DCFH-DA probe. After SDT treatment, the cells were cultured in the dark for additional 2 h before ROS analyses. As shown in Fig. 6a, the fluorescence intensity of DCF (green) in HCT116 and RKO cells after treatment with DVDMS alone was not significantly different from that reported for the control group. A slight increase in fluorescence was observed in some cells from the US group. In contrast, a large area of HCT116 and RKO cells from the SDT group showed a significant increase in fluorescence, indicative of intravascular ROS generation after SDT. This result was further confirmed by the histograms of DCF fluorescence intensity in cells, as detected by flow cytometry (Fig. 6b). For HCT116 cells in the control and DVDMS groups, only 2.31 ± 0.64% and 1.82 ± 1.78% of cells showed high DCF fluorescence, while the exposure to ultrasound alone increased the ratio to 8.53 ± 1.73%. Notably, the ratio of cells with high DCF fluorescence in the SDT group showed a significant increase to 41.50 ± 4.95%. RKO cells showed similar trend with HCT116 cells in the control, DVDMS and US groups. However, the ratio of cells showed high DCF fluorescence in SDT group was up to 48 ± 7.25% (*p* < 0.01). These results demonstrate the involvement of ROS in mediating SDT effects.

## Discussion

In general, the treatment of CRC involves surgical resection followed by chemotherapy and/or radiotherapy or immunotherapy [22]. However, safe resection with radio-chemotherapy is limited, owing to cancer metastasis or growth of chemotherapy-resistant tumors [23]. Although immunotherapy as an emerging therapy for CRC is promising, identification of an appropriate antigen target to prevent cytokine storm is still a great challenge [24]. Hence, efforts have been directed to tailor novel and effective treatment regimens owing to the poor prognosis of advanced human colon cancer using conventional methods. In this study, the cytotoxicity of DVDMS-mediated SDT has been investigated on two types of human-derived colorectal cancer cells (HCT116 and RKO), providing useful information toward this potential approach for application in CRC treatment.

SDT is a promising non-invasive anticancer therapeutic approach and relies on the cytotoxicity induced by the combination of a chemical sonosensitizer and ultrasound [17, 25–27]. In comparison with PDT, it offers the benefit of treating deep-seated tumors owing to the high penetration capability of ultrasound. The characteristic of the sensitizer is one of the key factors to determine the efficacy of SDT treatment. Majority of sonosensitizers are mostly derived from photosensitizers that have been widely used in PDT, which is unable to provide optimum treatment effect owing to unknown active ingredients and serious skin side-effects. With high purity and water solubility, DVDMS has relatively short-term skin phototoxicity and high sono-activity [13, 14, 19], suggestive of its potential as a favorable sonosensitizer. Accumulating evidence have shown that DVDMS-mediated SDT can effectively induce killing effect in multiple tumor cells, including K562 leukemia cells [17], esophageal cancer ECA-109 cells [14], breast cancer [28], sarcoma [19], glioblastoma cells [18], etc. Although this study included only in vitro experiment, the cytotoxicity induced by DVDMS-mediated SDT was found on both the two types of human colorectal cancer cells, excluding cell line specific effect. In line with Wang’s study, DVDMS-mediated SDT induced about 20% cell death rate in CT26 cells at 4 h after the treatment [29]. Collectively, these results indicated certain efficiency of DVDMS-mediated SDT on colorectal cancer cells, providing convincing support for further investigations.

The uptake and accumulation of sensitizers in cancer cells play critical role in the effectiveness of SDT. Due to its water solubility, DVDMS was found to be easily internalized by colorectal cancer cells in vitro. Our flow cytometry results showed that the amount of intracellular DVDMS increased significantly with the time of incubation, also evidenced by fluorescence imaging. In line with our findings, Hu et al. also found rapid uptake and accumulation of DVDMS in colorectal cancer cells SW620 [17]. However, they found markedly lower fluorescence intensity in three types of normal healthy cell lines (peripheral blood mononuclear cells PBMC, spleen lymphocytes SPL and mouse embryonic fibroblast NIH3T3). In contrary, our study showed evident uptake of DVDMS by normal colon cells NCM460 in vitro likewise, probably owing to its water solubility. Thus, further investigation of in vivo distribution of DVDMS in both tumor and normal tissue is warranted. The probable accumulation due to enhanced permeability and retention effect of tumors should be explored. Combined with focused ultrasound, SDT may benefit from local focusing as well as deep penetration. Nonetheless, the therapeutic effect on the tumor periphery with infiltrating tumor cells and normal cells is also worth examined in further study.

It is important to analyze the possible mechanism underlying SDT-mediated cell killing. ROS is a product of aerobic metabolism linked to mitochondria damage [30]. Studies showed that the excessive generation of intracellular ROS may serve as one of the vital factors contributing to SDT-induced cell damage [31]. It was reported that ALA-SDT induced osteosarcoma UMR-106 cell apoptosis both in vivo and in vitro through an ROS-related mitochondrial pathway [27]. Another study confirmed significant increase in the level of ROS after DVDMS-SDT, resulting in cellular apoptosis [32]. In this study, microscopic imaging and flow cytometry analyses showed significantly increased level of intracellular ROS after 2 h post-treatment with SDT, confirming the involvement of ROS in SDT-mediated cell killing.

Aside from only in vitro results incorporated, another limitation of this study is the moderate killing effect induced by the therapeutic parameters we used. Consistent with our results, the cell viability of murine colorectal cancer cells CT26 decreased moderately either [29]. Currently, a paucity of studies attempted to encapsulate DVDMS into liposomes, aiming to achieve better antitumor effect [33, 34]. Sun and Wang et al. designed DVDMS-encapsulating liposomes modified with a tumor-homing peptide iRGD (iRGD-Lipo-DVDMS). The cell viability of glioma cells C6 incubated with iRGD-Lipo-DVDMS was only about half of that with free DVDMS after exposure ultrasound [34]. Subsequent in vivo study confirmed the profound anti-glioma efficacy by SDT with iRGD-Lipo-DVDMS. Using microbubbles as the carrier, Li et al. designed a complex constituting from DVDMS-liposome with microbubbles via biotin–avidin linkage, which was called DLMBs. Compared with free DVDMS or DVDMS-liposome, it was proved that DLMBs exerted better antitumor activity on breast cancer in both in vitro and in vivo studies of SDT [33]. These studies provided promising strategies for improving the therapeutic effect of DVDMS-mediated SDT on tumor treatments.

## Conclusions

Our study investigated the synergistic antitumor effects of ultrasound and DVDMS against human colon cancer cells in vitro. DVDMS, as an alternative sonosensitizer, was activated by ultrasound and consequently induced cellular apoptosis. Moreover, intracellular ROS levels obviously increased during SDT. These findings demonstrated the antitumor effect of DVDMS-SDT related to ROS production, which could be further investigated in in vivo models for the treatment of human colon cancer.

## Methods

### Cell culture

Human colon cancer cell line HCT116 and RKO, and normal colon cell line NCM460 purchased from the Cell Bank of Type Culture Collection of the Chinese Academy of Sciences (Shanghai, China) were used in this study. HCT116 and RKO cells were cultured in Dulbecco’s modified Eagle’s medium (DMEM) supplemented with high glucose (HyClone, Logan, UT, USA), while NCM460 cells were cultivated in Roswell Park Memorial Institute (RPMI)-1640 medium (GIBCO, Invitrogen, Carlsbad, CA, USA). Both media were supplemented with 10% fetal bovine serum (FBS; BI, Biological Industries, Israel) and 1% penicillin and streptomycin (GIBCO, Invitrogen, Carlsbad, CA, USA). Cells were maintained in an incubator at 37 °C under a humidified atmosphere with 5% CO_2_. Cells at the exponential growth phase were used for all experiments.

### DVDMS

**Fig. 7:**
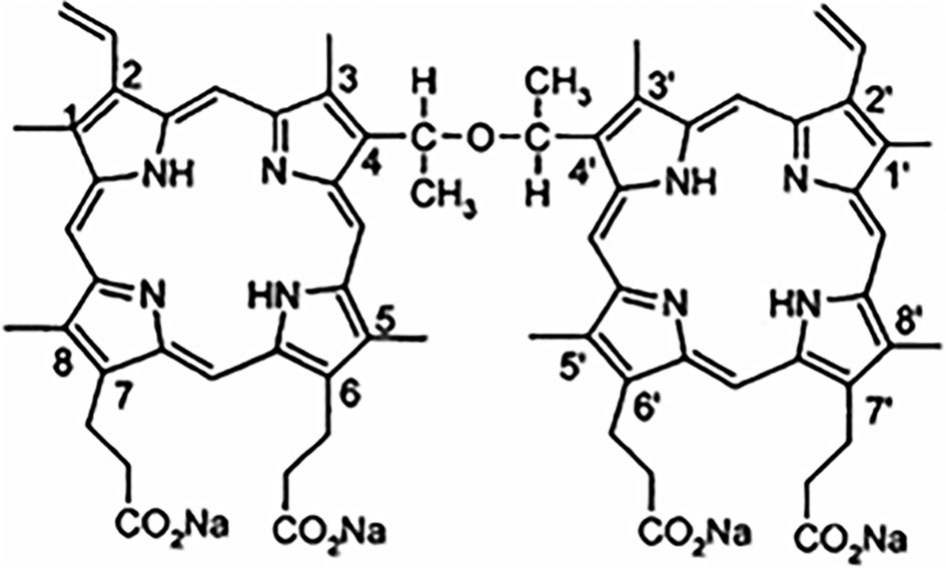
Chemical structure of DVDMS

DVDMS (molecular formula: C_68_H_66_N_8_O_9_Na_4_, molecular weight: 1230.265) with purity as high as 98.5% was generously provided by Jiangxi Qinglong Hi-tech Co., Ltd (Jiangxi, China). DVDMS was dissolved in phosphate-buffered saline (PBS, pH 7.2, BioScience, Shanghai, China) at a storage concentration of 2 mg/mL and sterilized using a 22-µm flitter (Jet Bio-Filtration Co., Ltd., Guangzhou, China), followed by storage in the dark at − 20 °C prior to use. The chemical structure of DVDMS is shown in Fig. 7.

### Spectral analysis of DVDMS

The absorption spectra of DVDMS at 10 µg/mL concentration in PBS were recorded between 300 and 700 nm wavelength using an ultraviolet spectrophotometer system (G6860A, Agilent Technologies, Malaysia), while its emission spectra at various concentrations (5, 10, 20, 30, and 40 µg/mL) in PBS were recorded using a microplate reader (BioTek, Winooski, VT, USA) under an appropriate excitation wavelength at 37 °C.

### Cytotoxicity assessment of DVDMS

HCT116, RKO and NCM460 cells were harvested and seeded in a 96-well plate at a density of about 1 × 10^4^ cells/well. After incubation for 24 h, the cells were treated with DVDMS at different concentrations (0, 1, 2, 5, 10, 15, and 20 µg/mL). Cell viability was analyzed 6 h post-incubation with DVDMS using CCK8 assay, which is based on that WST-8 (2-(2-methoxy-4-nitrophenyl)-3-(4-nitrophenyl)-5-(2,4-disulfonic acid benzene)-2H-tetrazole monosodium salt) is reduced by cellular dehydrogenases to an orange formazan product that is soluble in tissue culture medium. The amount of formazan produced is directly proportional to the number of living cells and is measured by absorbance at 450 nm. In brief, each well was treated with a mixture of 10 µL CCK8 solution (Solarbio, Beijing, China) and 90 µL serum-free medium after washing the cells twice with PBS, and the plate was incubated for 1 h at 37 °C. Optical density (OD) was measured at 450 nm using a microplate reader (BioTek, Winooski, VT, USA). Cell survival rate was calculated using the following equation:

$${\text{Cell survival }}\left( \% \right) \, = \, \left( {{\text{OD}}_{\text{treatment}} - {\text{ OD}}_{\text{blank}} } \right)/\left( {{\text{OD}}_{\text{control}} - {\text{ OD}}_{\text{blank}} } \right) \, \times { 1}00\% .$$

### Intracellular uptake of DVDMS

To analyze the intracellular localization of DVDMS in HCT116 cells and RKO cells, about 5 × 10^4^ cells/well were cultured in a glass-bottom culture dish. After 24 h, the cells were incubated with DVDMS (5 µg/mL) for 6 h, followed by gent washing with cold PBS twice and fixation with 4% paraformaldehyde for 20 min. The cells were stained with 1 µg/mL 4′,6-diamidino-2-phenylindole (DAPI) for 5 min and the intracellular fluorescence of DVDMS was observed under a laser scanning confocal microscope (LSM880, Carl Zeiss, Germany). To quantitatively investigate the effects of different incubation time points on the intracellular accumulation of DVDMS, the NCM460, RKO and HCT116 cells (2.5 × 10^5^ cell/well) were seeded into six-well plates and incubated with 5 µg/mL DVDMS for 0, 1, 3, 6, 9, and 12 h. The mean fluorescence intensity of intracellular DVDMS was analyzed using a flow cytometer (BD Accuri C6 Plus, BD Biosciences, Franklin Lakes, NJ, USA) and a microplate reader (BioTek, Winooski, VT, USA).

### Ultrasonic system and SDT protocol

**Fig. 8:**
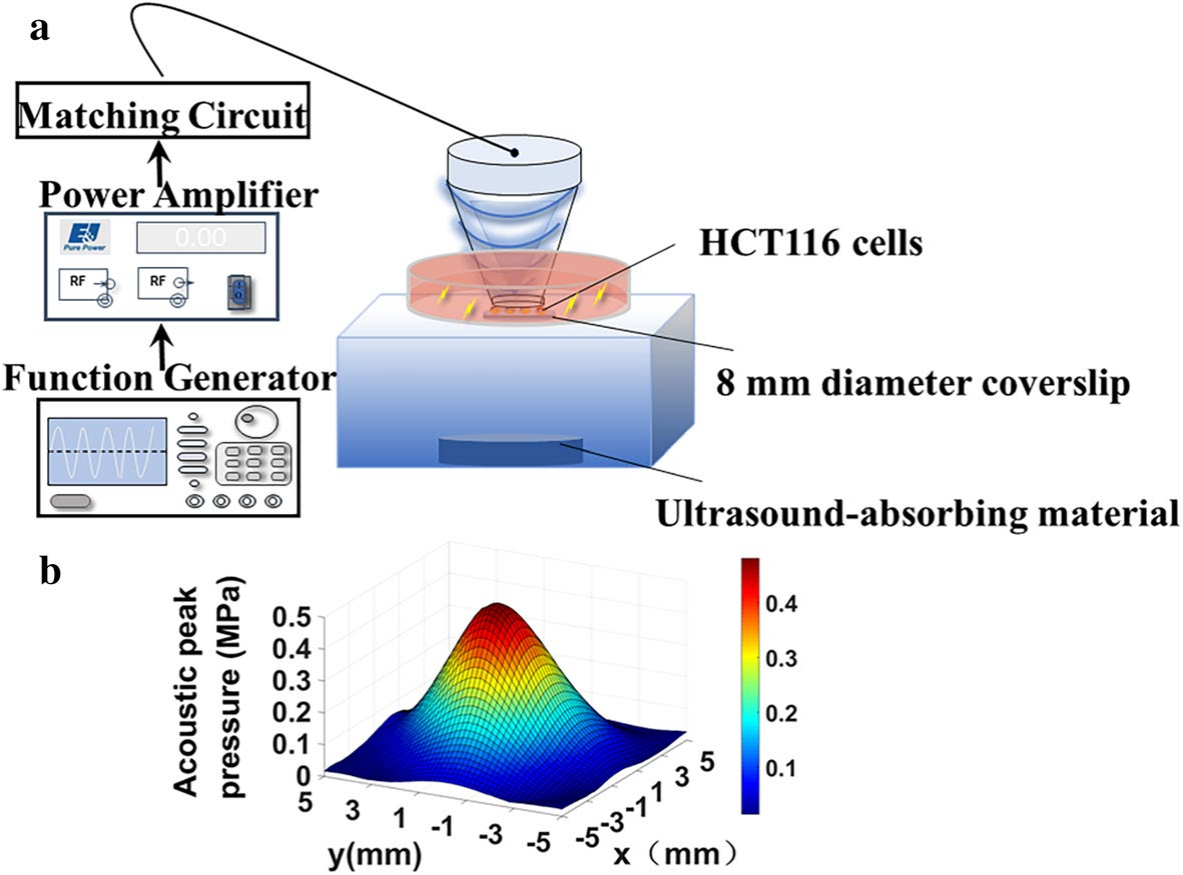
**a** Graphical illustration of the ultrasonic setup and **b** acoustic map of peak rarefactional pressure measured at the focal area in the lateral plane

The experimental apparatus used in this study is shown in Fig. 8. A homemade single-element spherical transducer (center frequency: 0.970 MHz, lateral and axial full-width at half-maximum intensity of the beam: 3.5 and 15 mm, respectively) was used, and the acoustic peak rarefactional pressure map at the focal area in the lateral plane was measured using a needle hydrophone (HNR-0500, Onda Corp, Sunnyvale, CA, USA). The transducer was driven by a 50-dB power amplifier (2100L, Electronics & Innovation, Rochester, N.Y., USA), which was connected to a functional generator (AFG3102C, Tektronix, Inc., Beaverton, O.R., USA) to produce ultrasound waves. The transducer was seamlessly immersed in a cone filled with degassed water. The cone tip was sealed with a thin polyurethane membrane, and the focal zone of the ultrasound beam was left 2 mm beneath the cone tip. Cells (8 × 10^4^ cells/slip) were seeded into an 8-mm-diameter circular coverslip. During the treatment, the coverslip was placed in the center of a cell culture dish filled with degassed water. The cone tip was immersed and placed at a distance of 2 mm above the coverslip to ensure that the cells were in the focal zone of the acoustic field. The cell culture dish was placed above a water tank filled with degassed water and a piece of ultrasound-absorbing material at the bottom.

The study had four groups as follows: a control group without any treatment (Control), group incubated with DVDMS alone for 6 h (DVDMS), group treated with ultrasound alone (US), and a group treated with SDT using DVDMS (SDT). The ultrasonic parameters used in US and SDT groups were the same (center frequency: 0.970 MHz; acoustic power: 3.45 W; duration: 3 min; duty cycle: 30%). For each group, cells on five coverslips were used to undergone the treatment and collected for further flow cytometry analysis. The treatments were repeated five times. The cell coverslip was placed slightly at the bottom of the cell culture dish and exposed to ultrasound. Cellular apoptosis and necrosis were evaluated after different treatments.

### Analyses of cellular apoptosis and necrosis

Cells were seeded (8 × 10^4^ cells/well) on circular coverslips placed in a 24-well plate. Cells were randomly divided into four groups (Control, DVDMS, US, and SDT) as mentioned above. The cells in DVDMS and SDT groups were incubated with DVDMS (5 µg/mL) in the dark for 6 h. Then, the cells in US and SDT groups were exposed to ultrasound for 3 min. After treatments, cells were cultured for 2 h before flow cytometry analyses. Cellular apoptosis and necrosis were analyzed with an Annexin V-FITC and PI detection kit (BD Biosciences, San Diego, CA, USA). In brief, the cells were harvested and suspended in a flow tube using 200 µL 1× binding buffer. To adjust the fluorescence compensation, a blank control group (without staining) and two groups stained with PI or FITC solution separately were prepared. The cells in the four groups (Control, DVDMS, US and SDT) were stained with both FITC and PI solution. After staining in the dark, the apoptosis and necrosis of cells were immediately evaluated with flow cytometry (BD Accuri C6 Plus, BD Biosciences, Franklin Lakes, NJ, USA) using the FL-1 filter (excitation 488 nm, emission 525 nm) and FL-2 filter (excitation 488 nm, emission 590 nm).

### Detection of intracellular ROS after treatment

The probe 2,7-dichlorodihydrofluorescein diacetate (DCFH-DA) is a non-fluorescent and cell-permeable agent that is easily hydrolyzed to its de-esterified product DCFH, which could be oxidized into its highly fluorescent form 2,7-dichlorofluorescein (DCF) by intracellular ROS. Therefore, the fluorescence intensity of DCF is used to probing the intracellular ROS level. For qualitative analysis, HCT116 or RKO cells (8 × 10^4^ cells/well) were seeded on circular coverslips placed in 24-well plates. After incubation for 24 h, cells were treated with 10 μM DCFH-DA (Solarbio, Beijing, China) for 20 min at 37 °C with gentle shaking in the dark and then were divided into the four groups. At 2 h post-treatment, cells were subjected to fluorescence imaging or flow cytometry analysis. For fluorescence imaging, cells were carefully washed twice with PBS and then the fluorescence of DCF was observed using an inverted fluorescence microscope (ECLIPSE Ti, Nikon, Tokyo, Japan) with excitation and emission wavelengths of 498 and 522 nm, respectively. To quantify the fluorescence intensity of DCF, cells were harvested, washed with PBS, filtered, and analyzed immediately with flow cytometry (BD Accuri C6 Plus, BD Biosciences, Franklin Lakes, NJ, USA).

### Statistical analysis

All data are expressed as mean ± standard deviation, and statistical significance was determined using one-way analysis of variance (ANOVA). A value of *p* < 0.01 was considered statistically significant.

## Data Availability

The datasets used and analyzed during the current study are available from the corresponding author on reasonable request.

## Abbreviations

SDT: Sonodynamic therapy
DVDMS: Sinoporphyrin sodium
CRC: Colorectal cancer
PDT: Photodynamic therapy
ROS: Reactive oxygen species
CCK8: Cell Counting Kit 8
FITC: Fluorescein isothiocyanate
PI: Propidium iodide
DAPI: 4′,6-Diamidino-2-phenylindole
DCF: Dichlorofluorescein
DCFH-DA: 2,7-Dichlorodihydrofluorescein diacetate
ALA: 5-Aminolevulinic acid
OD: Optical density
AlPcTS: Chloroaluminum phthalocyanine tetrasulphonate
